# GC–MS analysis of soil faecal biomarkers uncovers mammalian species and the economic management of the archeological site "Le Colombare di Negrar"

**DOI:** 10.1038/s41598-023-32601-9

**Published:** 2023-04-04

**Authors:** Chiara Reggio, Erika Palmisano, Umberto Tecchiati, Alessandro Ravelli, Roberta F. Bergamaschi, Paola Salzani, Cristiano Putzolu, Sara Casati, Marica Orioli

**Affiliations:** 1 Dipartimento di Scienze dell’Antichità, Sapienza Università di Roma, Rome, Italy; 2 Dipartimento di Scienze Biomediche, Chirurgiche ed Odontoiatriche, Laboratorio di Tossicologia Forense, Università degli Studi di Milano, Milan, Italy; 3 Dipartimento di Beni culturali e ambientali, PrEcLab–Laboratorio di Preistoria, Protostoria ed Ecologia Preistorica, Università degli Studi di Milano, Milan, Italy; 4 Ministero della Cultura, Soprintendenza archeologia, belle arti paesaggio per le Province di Verona, Rovigo e Vicenza, Verona, Italy; 5 Dipartimento di Storia Culture Civiltà, Alma Mater Studiorum Università di Bologna, Bologna, Italy

**Keywords:** Analytical chemistry, Environmental social sciences

## Abstract

The identification of the mammalian species based on faecal sediments in modern and ancient environments is the aim of the research of archaeologists, forensic scientists and ecologists. Here, we set up and validated an optimized gas chromatography-mass spectrometry (GC–MS) method, characterized by a time-saving sample preparation protocol, for the simultaneous analysis of faecal biomarkers (6 sterols/stanols and 5 bile acids) in 14 soil samples from the archaeological site of “Le Colombare di Negrar” in northern Italy. Although the archaeological sediment samples examined are numerically exiguous, a comparative reading of our faecal biomarkers findings with new studies on faunal materials collected in the same stratigraphic detail during recent excavation campaigns will allow to better clarify the economic interest of the animal species farmed in the Colombare site (such as bovines, goats, sheep and pigs) and to shed light on the management of breeding. Together with archaeozoological and archaeobotanical analyses, the investigation of faecal biomarkers can increase our knowledge of how ancient local communities exploited natural resources and may allow us to deduce what their impact on the landscape was.

## Introduction

**Figure 1:**
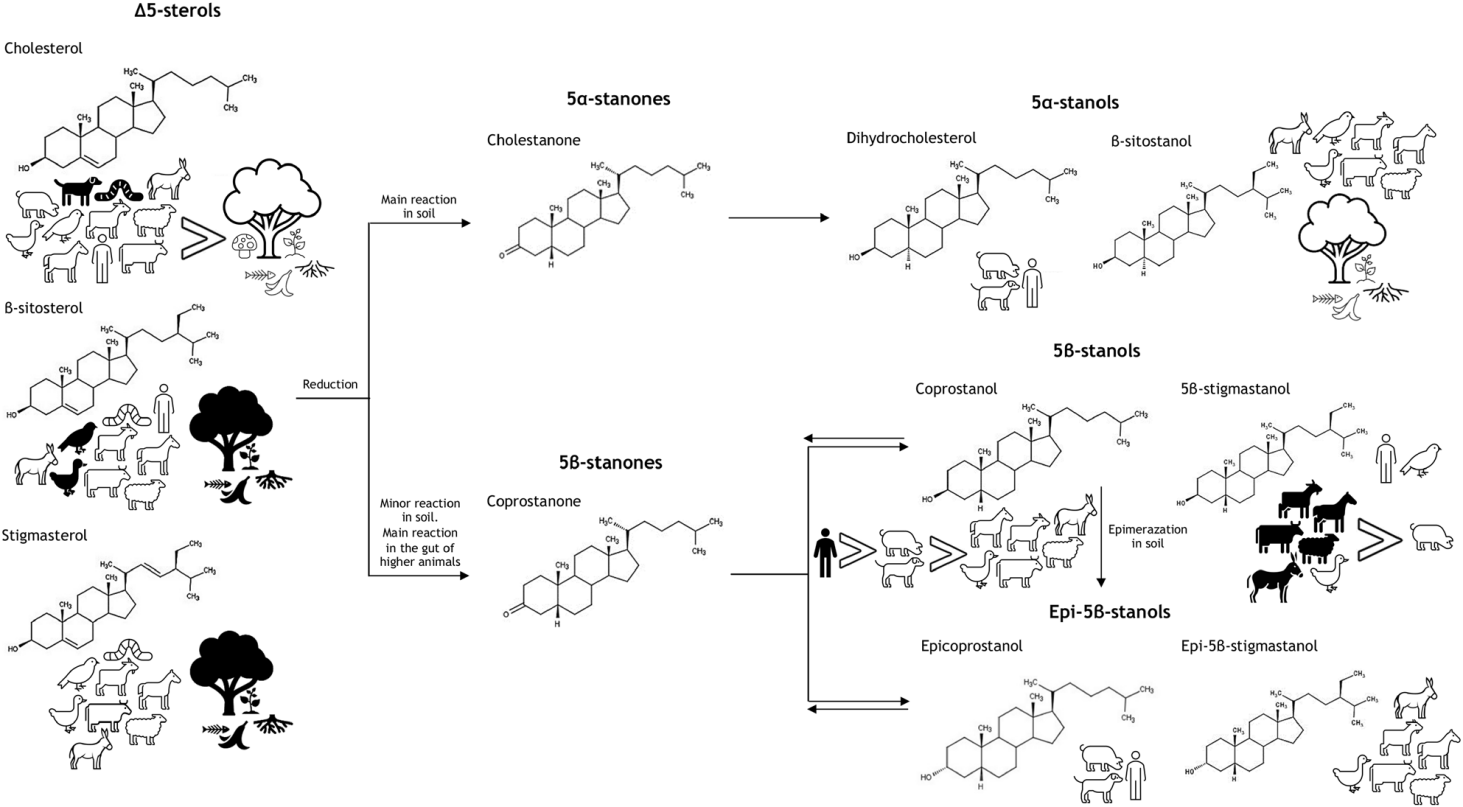
Δ5-sterols, stanols and stanones in the environment: prevalent compounds in black pictures (modified from Prost et al.).

**Figure 2:**
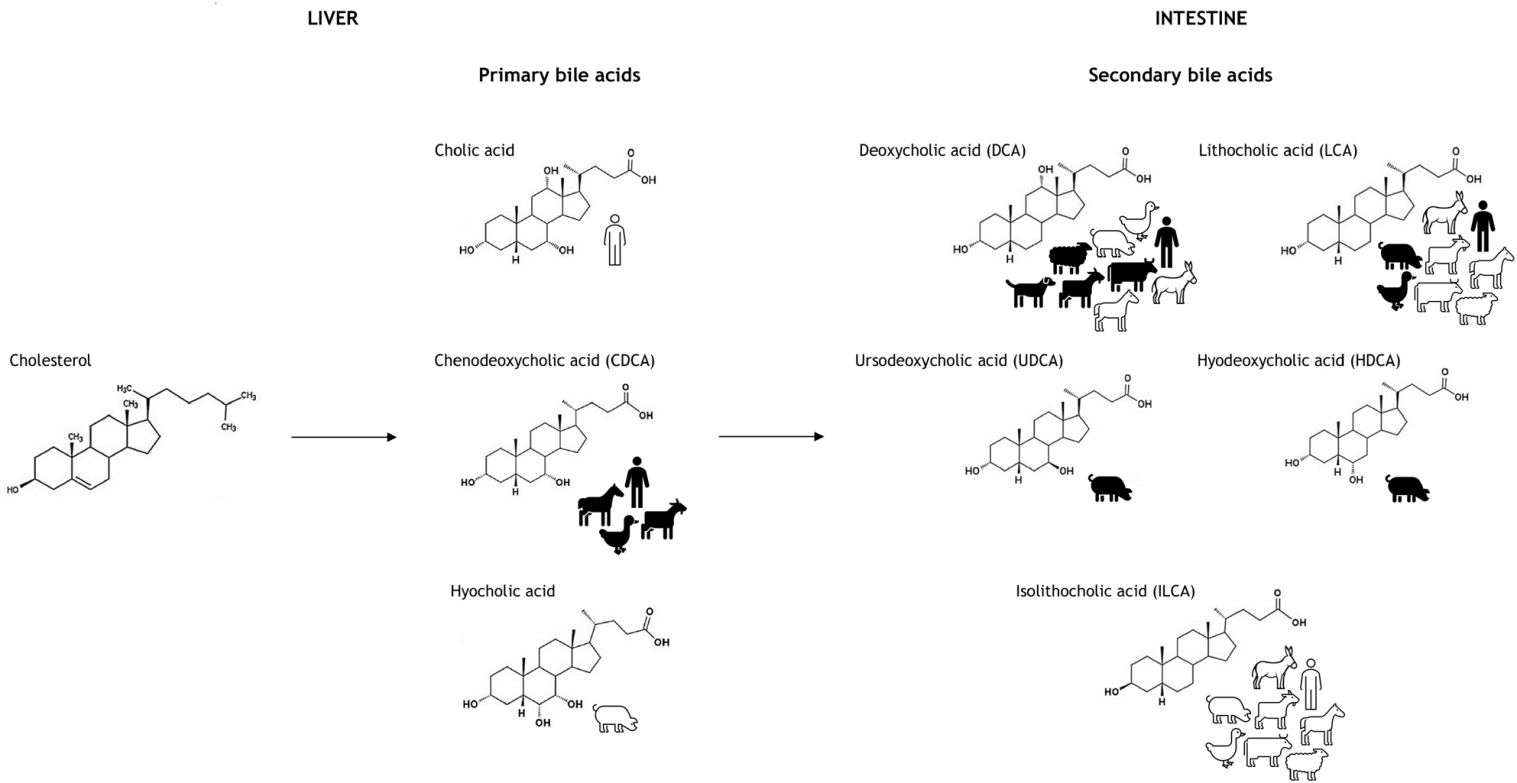
Primary and secondary bile acids: prevalent compounds in black pictures (modified from Prost et al.).

Dung is a matter present in archaeological deposits much more than we could think, but it is rarely macroscopically recognizable. Thus, the search for dung in the soil using peculiar chemical markers can demonstrate its presence in the archaeological record, even in the absence of fossil traces. Then, once the faecal matter is detected, the analysis of its chemical composition leads to the identification of the animal species that have produced it. The recognition of the defecator animal species seems to be one of the main prerequisites for further studies on dung deposits in archaeological sites^1^. Among the various components, some lipid molecules, belonging to the class of steroids, appear to be particularly useful as direct biomarkers of animal faeces. Indeed, steroid analysis is a promising tool if the archaeological context indicates a faecal input. This is mainly due to the high stability of these molecules, under a lack of oxygen, making them suitable for tracing a faecal input that occurred hundreds to thousands of years ago^2–6^. Furthermore, steroids (in particular 5β-stanols) show a low water solubility and the capacity to bind to particulate organic matter^7^. As a consequence, they don’t tend to leach but they bind to the soil matrix^7,8^. Among steroids, the classes of Δ5-sterols, stanols, stanones are the best candidates to act as biomarker of the species. Δ5-sterols are present in plants, fungi, animal (including human) tissues and faecal remains. In particular: Stigmasterol and β-sitosterol are the typical Δ5-sterols for plant biomass, whereas cholesterol is the dominating Δ5-sterol in most animal tissues^9,10^. However, cholesterol can be found also in plants (0–70% of total sterols), root exudates, as well as in several fungal species^9,11–13^. Thus, cholesterol as well as stigmasterol and β-sitosterol can be widespread in soil (Fig. 1). Stanols are mostly produced by microbial processes from Δ5-sterols. For example, the 5α-stanols, 5α-stigmastanol and 5α-cholestanol are formed in the environment from their sterol precursors, i.e. β-sitosterol and cholesterol^14–16^ (Fig. 1). However, small amounts of 5α-stanols have also been found in fresh plant and animal tissue^17,18^. On the other hand, 5β-stanols and epi-5β-stanols are mainly produced by specialized microorganisms in the gut of higher animals, but only to a lesser extent in the environment^15,19–22^ (Fig. 1). Stanones have seldom been analyzed yet^23,24^. They are formed during the transformation of Δ5-sterols to 5β-stanols, 5α-stanols, and epi-5β-stanols, both in the gut of higher animals as well as in the environment^15,19,25^ (Fig. 1). Hence, Δ5-sterols, stanols, and stanones reach the soil not only via faeces, but also via dead plant or animal material, or root exudates, or soil flora and fauna; in addition, they are directly formed in soil by microorganisms from precursor sterols (Fig. 1). The fact that Δ5-sterols are ubiquitarian, besides their transformation to stanols in the environment, make them not suitable to be used as faecal biomarkers. In contrast, 5β-stanols and 5β-stanones, and epi-5β-stanols, being mainly produced in the gut of higher animals, might be better candidates. The stanols profile in faecal material can be related to a particular mammalian species on the basis of its diet (main sterol uptake), its ability to biosynthesize endogenous sterols (secondary sterol uptake) and the way it metabolizes sterols and converts them into stanols with the help of intestinal flora^26^. Looking into the stanols profile, we know that high levels of cholesterol-derived 5β-stanols are found in the faeces of omnivores and carnivores (coprostanol and epicoprostanol), while 5β-stanols derived from β-sitosterol, a phytosterol^5^ in herbivore faeces (24-ethylcoprostanol and 24-ethylepicoprostanol)^27^. To gain more specificity, several ratios between 5β-stanols as well as their transformation products have been proposed to date^28,29^. Several studies^15,27,28^ pointed out that it is also essential to consider the steroid composition from soils nearby that had not received any faecal input (control samples) in order to be able to trace faecal inputs even when certain threshold values for steroid ratios fail to indicate so (e.g. by comparing steroid ratios of the soils with those of the control^2^). The only use of stanols fingerprint is nowadays not enough to identify whether the genus or species. An improvement in this challenge is represented by considering the ratio between 5β-stanols, in combination with the analysis of another group of faecal steroids, bile acids (BAs). BAs most likely represent the most specific markers for a faecal input, due to their exclusive occurrence in vertebrate faeces^30,31^. Furthermore, BAs are more stable to degradation than Δ5-sterols, stanols, and stanones^32^ and this feature makes them able to reveal an ancient faecal input into soils where other markers can’t because of their instability^4,23^. The primary BAs are cholic acid and chenodeoxycholic acid in humans and chenodeoxycholic acid and hyocholic acid in pigs. They are produced in the liver from cholesterol, excreted into the intestine and then transformed by microbial activity to secondary BAs (Fig. 2)^15^. In the human body cholic acid, chenodeoxycholic acid (CDCA), and deoxycholic acid (DCA) return to the liver, whereas most of the secondary BA lithocholic acid (LCA) is excreted in faeces^33^. Due to different BA composition and metabolism, BA profiles of vertebrates (including humans) may differ significantly^15,33,34^. For example, Prost et al. proposes a DCA/LCA ratio of 5–48 for bovines, sheep and goats, while a DCA/CDCA ratio of 0.8–2.1 for horses^35^. Moreover, in goose faeces the content of DCA was 20 times smaller than CDCA content, whereas in goat faeces DCA was 30 times higher than CDCA. The first assessment of Δ5-sterols, 5α-stanols, 5β-stanols, epi-5β-stanols, stanones, and BAs in faecal samples from old livestock breed was performed by Prost et al.^35^. In this work, by using a combination of stanol and BA analysis together with the application of existing biomarker ratios, it was possible to distinguish between different faecal inputs for archaeological samples. We then follow the path of the work by Prost, with the aim of setting up an optimized GC–MS method, for the analysis of 6 sterols/stanols and 5 BAs. Our time saving method, being characterized by a shorter sample preparation, was then applied to the analysis of 14 different soil samples from the archeological site of Le Colombare di Negrar, in northern Italy.

## Materials and methods

### Chemicals and reagents

The reference standards cholest-5-en-3β-ol (cholesterol), 5-stigmasten-3β-ol or 24-ethyl-5-cholesten-3β-ol (β-Sitosterol), stigmasta-5,22-dien-3β-ol (stigmasterol), 5α-cholestan-3β-ol or 5α-cholestanol (dehydrocholesterol, DHC), 24α-ethyl-5α-cholestan-3β-ol (β-sitostanol), 5β-cholestan-3α-ol (epicoprostanol), 3α-Hydroxy-5β-cholanoic acid (lithocholic acid, LCA), 3α,12α-dihydroxy-5β-cholanoic acid (deoxycholic acid, DCA), 3α,7α-dihidroxy-5β-cholanoic acid (chenodeoxycholic acid, CDCA), 3α,6α-dihydroxy-5β-cholanoic acid (hyodeoxycholic acid, HDCA), 3α,7β-dihydro-5β-cholanoic acid (ursodeoxycholic acid, UDCA) were purchased as powder from Sigma Aldrich (Milan, Italy). The reference standards lithocholic acid-d4 and 17β-hydroxy-17-methylandrosta-1,4-dien-3-one (methandienone)^36^ were purchased as methanolic solution (100 µg/ml) and powder (100 mg), respectively, from Sigma Aldrich (Milan, Italy). Methanol (MeOH) for HPLC (≥ 99.9%) and MeOH for LC/MS were purchased from Merck KGaA (Darmstadt, Germany). N-hexane and dichloromethane (DCM) were of analytical grade and purchased from Carlo Erba (Milan, Italy). Buffer solution at pH 4.60 was purchased from Nova Chimica Srl (Milan, Italy). Hydrochloric acid (37%) was purchased from Panreac Química SAU (Barcellona, Spain). The derivatizing agent N,O-Bistrifluoroacetamide was acquired from Uct Specialties, LLC (Bristol, PA, USA).

### Soil sampling

Soil samples were collected from 2020 (*n* = 7) to 2021 (*n* = 7) at the archaeological site of “Colombare di Negrar di Valpolicella”. Soil samples collected during the 2020 excavation campaign (sample 1 to 7) were dated from 4325 to 3659 B.C. (sample 6 and 7) and from 3957 to 3532 B.C. (sample 2, 4 and 5). The date of origin about sample 1 and 3 is not available. Regarding the 2021 excavation campaign (sample 8 to 14), the radioactive dating is still ongoing. However, the stratigraphic units of all soil samples are available in Table 1.

**Table 1:** Soil samples collected during 2020 and 2021 excavation seasons from the archaeological site of “Colombare di Negrar di Valpolicella” (SU: stratigraphic unit).

Year	Soil samples	SU
2020	1	US4 (US3)
	2	US3
	3	US4
	4	US3
	5	US3
	6	US6
	7	US6
2021	8	US1
	9	US4
	10	US5
	11	US6
	12	US7
	13	US8
	14	US9

**Figure 3:**
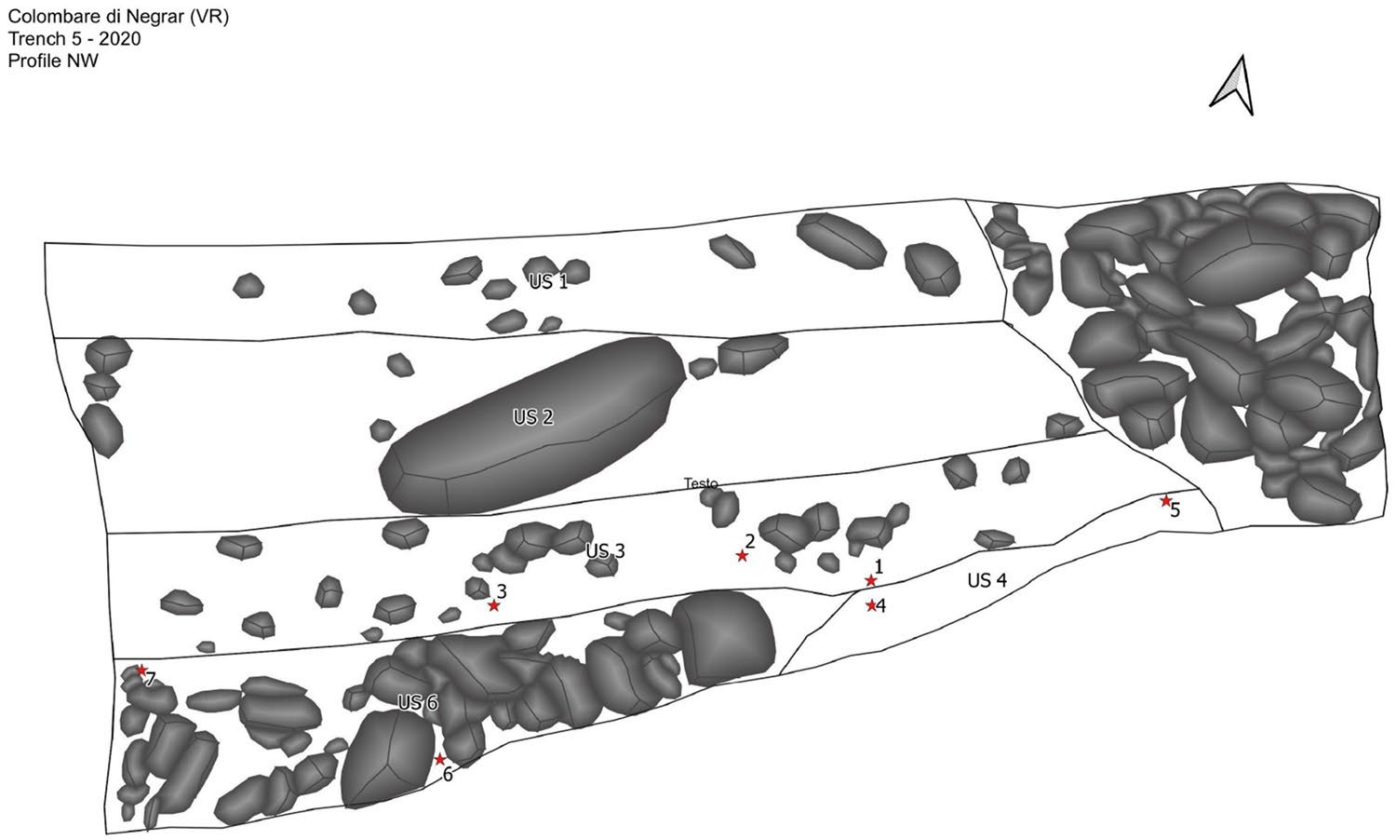
Sediment fractions related to the 7 samples of the 5–2020 trench (US4 (US3): sample 1; US3: sample 2; US4: sample 3; US3: sample 4; US3: sample 5; US6: sample 6; US6: sample 7).

**Figure 4:**
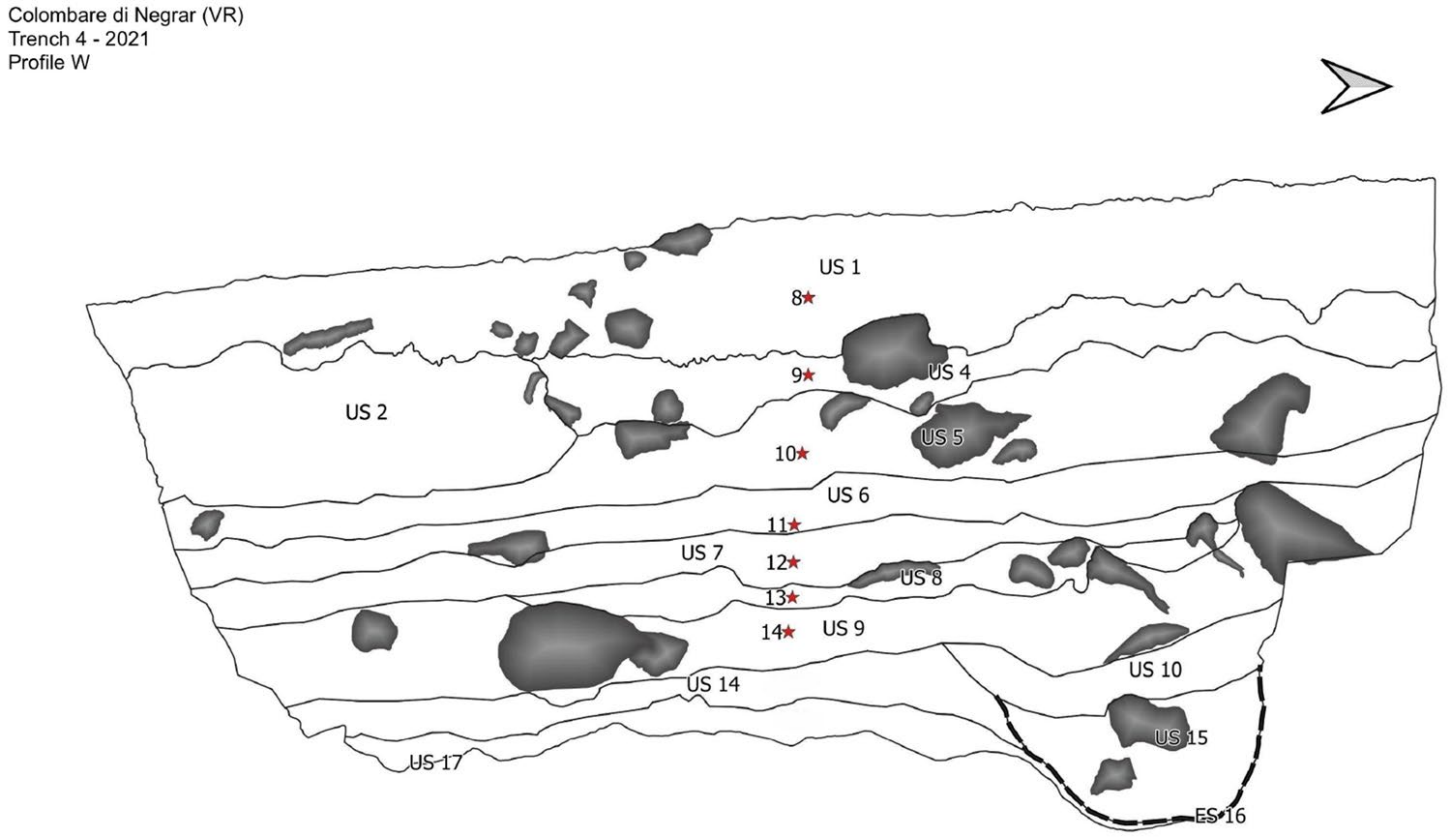
Sediment fractions related to the 7 samples of the 4–2021 trench (US1: sample 8; US4: sample 9; US5: sample 10; US6: sample 11; US7: sample 12; US8: sample 13; US9: sample 14; US14: sterile layer).

In addition, soil samples were collected from the same sequences from which the pollen samples and the animal residues dated with C14 were taken^37^. Specifically, samples 1–7 come from trench 5 in 2020 (Fig. 3), which is to be considered as describing older time horizons compared to the remaining samples 8–14, which belong to trench 4 in 2021 (Fig. 4). The latter investigated stratigraphic units comparable to the trench 5–2020 and more recent sediments. Samples 6–7 of the US 6 (the deepest stratigraphic unit found in the 5–2020 trench) date in a time range between 4325 and 3659 cal BC (95.4% probability), while samples 2–4-5 of the US3, are dated in the range between 3957 and 3532 cal BC (95.4% probability)^37^.

The sediment fractions taken for faecal biomarker analyses were collected in the stratigraphic section (Table 1) at the same sampling points as those for palaeobotanical analyses^37^. Sampling was performed by a 5 cc sterile syringe horizontal motion from the outside to the inside namely “carrot model” using sterile gloves to avoid contamination from the skin. The syringes were then vacuum sealed and kept at − 20 °C. Steroid-free soil samples were collected from a sterile layer (this one, located at the bottom of the stratigraphic sequence, corresponds to the soil layer that overlaid the rocky substrate and was deposited in times before the setting of the prehistoric village, therefore it is devoid of traces of human activities) using the same procedure and analysed to exclude any source of chromatographic interferences. Blank soil was used for calibrators and quality controls (QCs). All samples were sent to our laboratory and kept at − 20 °C until analysis.

### Sample preparation

For each sample obtained as explained in "Soil sampling" section, 1 g of soil was added with 25 μL of LCA-d4 (10 γ/mL) and with 25 μL of methandienone (10 γ/mL) as IS for BAs or sterols and stanols, respectively. 1 mL of DCM and 0.5 mL of MeOH were added to samples, which were then ultrasonicated at room temperature for 30 min. The sample was centrifuged at 3095*g* for 10 min. The supernatant was transferred in Eppendorf of 1.5 mL and centrifuged in an ultracentrifuge at 17,864*g* for 10 min. The clear supernatant was then transferred into glass test tubes and dried under a stream of nitrogen. The dried residue was reconstituted with 1 mL of buffer solution at pH 4.6 and then added with 300 μL of HCl 0.125 N and 4 mL of n-hexane/ethyl acetate (9:1; v/v). After centrifugation, the organic layer was separated and dried under a stream of nitrogen. The dried residue was derivatized with BSTFA at 75 °C and 2 μL aliquot was injected into GC/MS system for the analysis.

### Equipment

GC/MS analysis was performed on an Agilent 6890N gas chromatograph interfaced with a single quadrupole 5973N detector (Palo Alto, CA, USA). The GC separation was carried out on an Agilent capillary column Varian CP-Sil8 (15 m length × 0.25 mm i.d., 0.25 μm film thickness) using the following oven temperature program: from 70 °C (held 2 min) to 160° at 30 °C/min, then to 300 °C at 15 °C/min (held 4 min). The injector temperature was 270 °C, the ion source temperature was 230 °C; carrier gas (helium) flow was 1.1 mL/min; the injection mode was splitless; the injection volume was 2 μL; the run time was 18.33 min and the mass spectrometer mode was electron ionization conditions (70 eV) by selected ion monitoring (SIM) mode. The characteristics fragment ions are showed in Table 2. Data acquisition and processing were performed using Agilent Chemstation (Palo Alto, CA, USA).

**Table 2:** Retention time, molecular weight and mass spectral characteristics of BAs, sterols and stanols (analyzed as TMS derivatives).

Compound	Retention time (min)	Molecular weight (g/mol)	Characteristic Fragment ions (m/z)		
			Target ion	Qualifier ion 1	Qualifier ion 2
LCA-d4	15.3	380.6	434.4	261.3	219.2
LCA	15.3	376.6	430.4	257.3	215.2
HDCA	15.8	392.6	345.3	428.4	255.2
UDCA	15.8	392.6	518.4	428.4	
DCA	16.4	392.6	428.4	345.3	256.2
CDCA	16.8	392.6	413.3	428.4	255.2
Methandienone	12.8	300.4	122.1	161.2	242.2
Epicoprostanol/coprostanol	14.3	388.7	370.4	355.4	215.2
Cholesterol	14.7	386.7	458.4	368.4	329.3
DHC	14.8	388.7	445.4	460.5	215.2
Stigmasterol	15.5	412.7	484.5	394.4	255.2
β-Sitosterol	15.9	414.7	486.5	396.4	357.4
β-Sitostanol	16.0	416.7	473.5	488.5	383.4

### Preparation of standard solutions, calibrators and quality control (QC) samples

Stock solutions of reference materials and internal standards (ISs) were stored in the dark at − 20 °C. Working solutions were prepared in MeOH from stock solutions at the following concentrations:

- sterols and stanols (cholesterol, β-sitosterol, stigmasterol, dihydrocholesterol, β-sitostanol, epicoprostanol, coprostanol) 1 μg/mL;
- BAs (LCA, DCA, CDCA, HDCA, UDCA) 1 μg/mL;
- LCA-d4 10 μg/mL;
- methandienone 10 μg/mL

and used for the preparation of calibration curves and QC samples.

Calibration standards (CS) and quality control (QC) for sterols, stanols and BAs were prepared from steroid-free soil (1 g) by adding working solutions to reach final concentrations of 5, 10, 25, 50, 125 pg/mg.

### Validation procedure

Method validation was carried out according to the current Eurachem guidelines^38^ and the following parameters were assessed: linearity, precision and accuracy, sensitivity in terms of limits of detection (LODs) and limits of quantitation (LOQ), specificity, recovery and carry-over.

#### Linearity

Calibration standards (n = 6) were obtained by spiking steroid-free soil with appropriate amounts of working solutions in the range 5–125 pg/mg as described at "Preparation of standard solutions, calibrators and quality control (QC) samples" section. The calibration curves were constructed by linear regression analysis of the peak area ratios of steroids to the ISs against nominal analyte concentration. The correlation was tested over the whole range of concentration (5–125 ng/mg). Linearity was considered satisfactory if r^2^ $\ge$ 0.990 and coefficient of variation $\le$ 15%.

#### Precision and accuracy

Intra-day precision and accuracy were determined at three concentration levels (i.e. 5, 25 and 125 pg/mg) through the analysis of six independent replicates of QCs. Precision and accuracy were estimated from the percent variation coefficient and the percent bias (bias%), respectively.

#### LOD and LOQ

The specificity of the analysis and matrix-to-matrix reproducibility were evaluated by comparing the GC/MS chromatograms of the analytes at the Limit of Quantification (LOQ) to those of the blank matrix. Sensitivity was expressed in terms of Limit of Detection (LOD) and Limit of Quantification (LOQ). The LOQ was determined as the lowest concentration with precision and accuracy values within ± 20% and a signal-to-noise (S/N) ratio of the peak areas ≥ 10, whilst the LOD was determined as the lowest concentration with a S/N ratio ≥ 3.

#### Extraction recovery

The extraction recovery (%) was calculated at the same three concentration levels selected for precision and accuracy by comparing the mass spectrometer responses obtained from free-steroids soil samples spiked, respectively, before and after the extraction step and expressed as percentage ratio between the two measured concentrations.

#### Carry-over

The injection of a blank non-spiked sample after the highest calibration level was used to evaluate the carry-over effect. It was considered negligible if the S/N ratio was lower than 3 at the analytes retention time.

## Results

### Optimization of sample preparation protocol

An optimized extraction procedure for steroids from sediments was set up starting from previous published protocols^39^. Specifically, several buffers (i.e. pH 4, pH 4.6, pH 9) and solvent combinations (i.e. N-hexane: ethyl acetate 9:1 v/v, N-pentane and chloroform:N-heptane:isopropanol 50:33:17 v/v/v) were tested in order to obtain a time and solvent saving single step extraction procedure. Maximum effectiveness was obtained using the mixture of N-hexane:ethyl acetate (9:1 v/v) as solvent after a pH adjustment at pH 4.6.

### Method validation

All calibration curves showed good linearity for all considered compounds over the entire investigated range when using a weighted 1/×2 linear correlation. All the results are reported in Table 3 together with the mean CV% and the calculated LOD and LOQ values. Precision data were considered satisfactory, because the CV% values lied below 20% for the low calibration level and below 15% for the others. Satisfactory accuracy results were also achieved, with experimental average concentrations lying within ± 15% from the expected value (Table 4). Likewise, the extraction recovery results were considered acceptable (Table 4). Finally, no carry-over effect was noticed, since a S/N *ratio* lower than 3 was observed in the blank sample injected after the highest calibration point for all the target compounds.

**Table 3:** Validation parameters.

Analyte	r2	CV (%)	LOD (pg/mg)	LOQ (pg/mg)
LCA	0.994	11.6	0.3	0.9
HDCA	0.991	14.8	0.4	1.5
UDCA	0.992	14.2	0.3	1.0
DCA	0.990	16.1	0.7	2.2
CDCA	0.992	11.6	1.3	3.5
Epicoprostanol/coprostanol	0.996	12.7	1.2	4.0
Cholesterol	0.997	23.3	1.3	4.5
DHC	0.995	7.5	1.4	4.8
Stigmasterol	0.993	23.5	1.0	3.3
β-Sitosterol	0.990	9.2	1.6	4.4
β-Sitostanol	0.994	21.2	0.9	2.9

**Table 4:** Intra-day precision, accuracy (bias%), and recovery for each compound.

Analyte	Precision			Accuracy (bias%)			Recovery (%)		
	5 pg/mg	25 pg/mg	125 pg/mg	5 pg/mg	25 pg/mg	125 pg/mg	5 pg/mg	25 pg/mg	125 pg/mg
LCA	10.2	6.8	0.8	12.2	6.5	0.9	41.8	32.4	40.9
HDCA	7.2	5.3	2.1	9.6	4.7	2.2	39.3	49.3	48.7
UDCA	11.7	9.5	2.1	15.5	8.7	2.2	30.8	35.5	37.8
DCA	10.1	12.8	4.7	11.1	12.4	4.6	27.0	35.0	36.5
CDCA	10.9	11.6	2.6	11.0	11.7	2.7	45.5	38.0	48.6
Epicoprostanol/coprostanol	12.7	4.8	1.4	14.2	4.8	1.4	44.1	48.3	38.8
Cholesterol	11.4	8.7	1.5	13.0	8.5	1.6	62.0	73.1	61.9
DHC	12.5	11.7	2.3	13.2	11.3	2.3	33.2	32.5	38.7
Stigmasterol	13.0	11.5	3.1	15.0	11.2	3.2	36.0	43.9	40.0
β-Sitosterol	9.9	9.6	4.7	11.9	8.8	4.8	53.1	85.3	61.8
β-Sitostanol	12.0	7.8	1.5	14.7	7.6	1.5	45.6	32.5	45.6

### Steroids quantification in sediment samples from the archaeological site of “Colombare di Negrar di Valpolicella”

**Figure 5:**
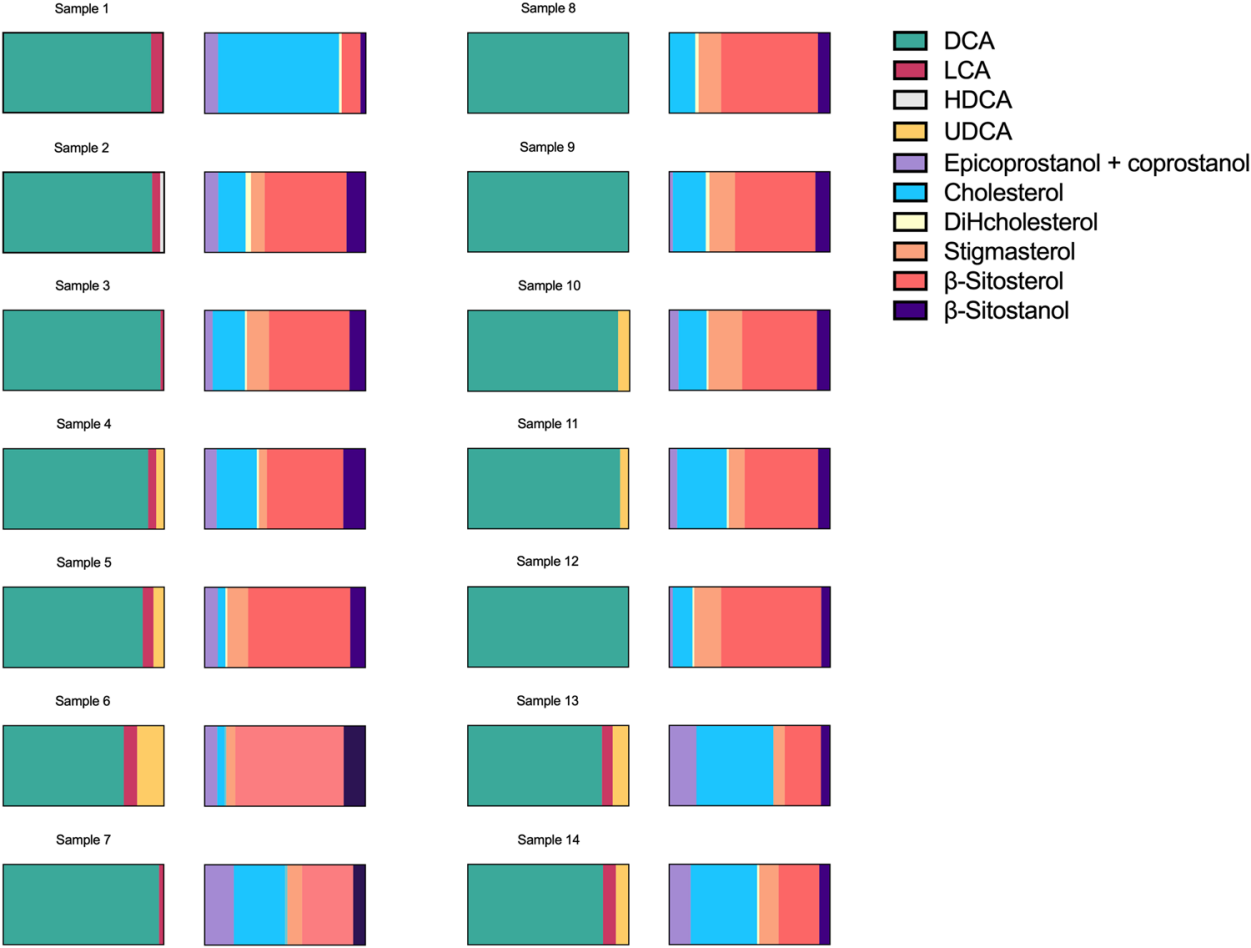
Results in % of BAs and steroids in soil samples 1–7 from the 2020 and 8–14 from 2021 excavation campaigns displayed as part of whole.

The optimized GC–MS method was applied to the analysis of 14 different soil samples from the archeological site of “Colombare di Negrar di Valpolicella”, in northern Italy. Results of BAs and steroids content in soil samples are summarized in Fig. 5 and in Tables 5 and 6. CDCA and HDCA were quantified > LOQ only in sample 2 and 12, respectively, while steroids were ubiquitous. To be mentioned that it was impossible, with our equipment, to separate epicoprostanol and coprostanol, since their identical r.t and fragments. For this reason, the peak area is relative to the sum of the two molecules.

**Table 5:** BA contents and ratios of analysed soil sediments.

Bile acids	Bile acids content (pg/mg dry matter)													
	1	2	3	4	5	6	7	8	9	10	11	12	13	14
Replicate	*n* = 2													
Year	2020							2021						
LCA	3.4	2.5	1.6	1.8	2.1	1.3	2.0	11.0	2.7	2.3	1.3	2.3	3.6	3.4
HDCA	< LOQ	1.8	< LOQ	< LOQ	< LOQ	< LOQ	< LOQ	< LOQ	< LOQ	< LOQ	< LOQ	< LOQ	< LOQ	< LOQ
UDCA	2.0	< LOQ	< LOQ	2.8	3.3	3.7	< LOQ	< LOQ	< LOQ	10.4	4.6	4.0	5.0	4.4
DCA	43.7	46.3	43.3	60.1	48.3	19.7	60.8	217.2	119.5	97.6	87.5	133.8	45.7	38.3
CDCA	< LOQ	< LOQ	< LOQ	< LOQ	< LOQ	< LOQ	< LOQ	< LOQ	< LOQ	< LOQ	< LOQ	3.4	< LOQ	< LOQ
DCA/LCA	12.7	18.5	27.7	33.6	22.7	15.7	29.9	19.7	43.5	41.7	67.9	57.3	12.6	11.2
DCA/CDCA	–	–	–	–	–	–	–	–	–	–	–	39.7	–	–
CDCA/LCA	–	–	–	–	–	–	–	–	–	–	–	0.7	–	–
HDCA/LCA	–	0.7	–	–	–	–	–	–	–	–	–	–	–	–

**Table 6:** Sterol and stanol contents of analysed soil sediments.

Steroids	Steroid content (pg/mg dry matter)													
	1	2	3	4	5	6	7	8	9	10	11	12	13	14
Replicate	n = 2													
Year	2020							2021						
Epicoprostanol + coprostanol	6.0	6.7	9.4	19.0	26.4	15.9	21.6	8.7	17.3	38.3	18.4	22.0	22.6	18.8
Cholesterol	52.3	12.5	36.3	66.0	16.1	7.6	39.7	539.5	174.5	117.5	118.9	135.3	62.4	55.6
DHC	1.1	2.7	1.7	3.0	2.4	1.8	2.8	75.4	16.4	8.9	2.5	8.0	0.2	1.0
Stigmasterol	0.4	6.3	23.4	12.0	40.1	11.0	10.4	495.6	142.6	137.6	39.1	182.2	9.5	15.6
β-Sitosterol	7.9	38.1	87.2	125.5	196.2	127.7	38.5	2072	438.2	312.6	179.7	703.0	29.2	33.5
β-Sitostanol	2.1	9.1	17.3	37.6	27.7	25.7	9.9	266.9	80.6	51.2	27.4	59.2	7.5	9.1

Moreover, BA ratios were calculated in order to distinguish between the different faecal inputs (Table 5).

## Discussion

### Steroid profiles of soil samples

Steroid biomarkers have been studied for both an identification of a faecal inputs into the soil, but also for a source assignment of the faecal input. In this work, we set up and validated the first GC–MS method able to identify and quantify (in the range 5–125 pg/mg) simultaneously both the BAs and the steroids in soil samples using a time and solvent saving extraction procedure. Rather than previously published protocols^39^, which required 36-h incubation and multiple liquid–liquid (LL) and solid phase (SP) extraction steps with large quantities of solvent, here we performed a 30-min incubation and a single LL extraction procedure. The optimized sample preparation allowed to also reduce the amount of soil from 5 to 10 g^39^ or 10^35^ to 1 g soil. Besides these improvements, our method presents a weakness, if compared to Birk et al.^39^_,_ that is the extraction recovery. For some analytes is quite low (around 30–40%) but for all of them is reproducible (CV < 0.25%). Moreover, the LOQ we obtained is totally enough for our purpose, so we can consider unnecessary a better extraction recovery.

Tables 5 and 6 and Fig. 5 present steroid and BA profiles of faeces from 14 samples collected at the archaeological site of “Colombare di Negrar di Valpolicella” during the 2020 and 2021 excavation campaigns.

All these samples have shown an elevated presence of β-sitosterol, followed by the other phytosterol stigmasterol. The large content of these two sterols, which are typical of vegetables, can be explained by the contribution from the surrounding vegetation, although pollen analyses show a prevalence of herbaceous species over tree species and thus a reduction in the forest already underway^40^. Since the data on the floristic composition indicate open places subject to frequent trampling, such as paths or pasture land, and the fungal spores detected suggest the local presence of domesticated herbivorous animals^40^, the content of the two sterols mentioned above can heuristically be associated with the possible decay of wooden structures set up on site or with plant material added to the soil and perhaps linked to foraging^41^. The identification of phytosterols (together with stanols and BAs) can suggest the existence of faecal inputs attributable to herbivore species^35^. According to Prost et al., a mostly vegetable-based diet suggests a content of Δ5-sterols, stanols and stanones ranging between 64 and 89% of the total phytosteroids and their bio-products. Except for sample 7, the results obtained from samples from US 6 and US 3 fell within these limits, which could provide support for this hypothesis.

The analysis of BAs, probably the most specific markers for faecal inputs, together with steroid ratios (see below), given their exclusive presence in faeces of vertebrates, revealed the same data obtained by Prost et al.^35^. In detail, we found presence of DCA >  > LCA in all our samples (predominating bile acid of ruminant and human faeces); the DCA levels of the 5–2020 trench samples fell in the range from 80 to 97% in relation to the total BA content.

We applied, where possible, specific BA ratios^35^ to all the trench 5–2020 samples, and we observed that the DCA/LCA ratios fell within the interval 13–30. This interval is comprised within the DCA/LCA range of 5–48, provided by Prost et al.^35^, which indicates the presence of bovine and ovine faeces. These results support what has so far emerged from archaeozoological studies performed in Colombare^42^.

It should be specified that the Colombare site was initially investigated in the 1950s—and then again at the turn of the 1960s and the 1970s—under the scientific direction of the Natural History Museum of Verona. With the exception of the opening of a small preventive archaeology survey in 2015, excavations were resumed in 2019 under the scientific direction of the University of Milan and the Cultural Heritage Office for the provinces of Verona, Vicenza and Rovigo^43^. As the results of new archaeozoological studies are still awaited, the study of the faunal remains collected during the excavations in the 1950s, conducted by Riedel in 1976^42^, is the only published one available. Riedel observes an animal population dominated by domestic species (around 90%) with a good representation of bovines and sheeps. Cattle make up 37.2% of the determinated faunal remains, while ovines make up 24.8%. Other BAs in the 5–2020 (Fig. 3) trench samples were not detectable, except for traces of HDCA in sample 2 and quantities of UCDA > LOQ in samples 1,4,5 and 6. These BAs are related to swine faeces. Additionally, we also found epicoprostanol, which reflects an omnivore diet^35^. Riedel supports the presence of pigs (26.1%) in the village of Colombare^42^; however, it is not always possible to distinguish pigs from boars based on bones findings. For such a remote period, it is also not possible to determine whether the domestication of the pig had been completed. Nor can it be ruled out that pigs were reared in the wild, with the risk of uncontrolled hybridization with boars. It is also true that pig-boar hybridisation has been sought over the centuries to 'refresh the blood' of farmed pigs^36^. In all the 5–2020 (Fig. 3) trench samples we detected cholesterol. This may indicate the contribution of fauna remains to the soil, since cholesterol and its bio-products, like dehydrocholesterol and epicoprostanol, are found in high concentrations in omnivores’ faeces^35^. Specifically, dog faeces show a high predominance of cholesterol, probably because of a diet with discarded human food. Such food is usually rich in cholesterol then expelled as it is via faeces by canine species, since they don’t have microorganisms able to turn Δ5-sterols into 5β-stanols during digestion. Our cholesterol findings suggest a possible presence of these animals at the prehistoric site, considering also that dog, whose domestication can be traced back to the Upper Palaeolithic^44^ has always been loyal companion to humans.

However, despite the hypothesis of faecal inputs attributable to canine species is credible, we cannot attribute all observed cholesterol only to them, given how easy it is to find this ubiquitous analyte. In fact, cholesterol is a component of almost all eucaryotic cells, both in the soil and in the faeces of omnivore animals such as pigs and humans. It is also because of the high percentages of cholesterol in some samples (1,3 and 7) together with the presence of its transformation products, epicoprostanol and dehydrocholesterol, that we cannot exclude with certainty faecal inputs attributable to humans. However, the absence of CDCA and non-dominant LCA levels^15^ lead us to discard the supposition of human ejections in an area that we are sure humans wished to live in. Finally, the absence of CDCA, which can be identified only in the faeces of horses, sheep, goose and humans^35^, further indirectly confirms the later spread of equine and poultry species in Northern Italy compared to the period of foundation and development of the Colombare site.

The radiocarbon dating of the 7 samples of the 4–2021 trench that we analysed is still ongoing. Except for samples 13 (US8) and 14 (US9), which are those from the deepest stratigraphic units (Fig. 4) and those with predominance of cholesterol, in all other samples (8–12) the main compounds identified are the β-sitosterol, followed by the β-sitostanol. The results of these compounds in samples 8–12 fall within the interval 64–89% described by Prost et al. for the total Δ5-sterols, stanols and stanones content in faeces of animals following a mostly vegetable-based diet^35^. These data allow us to confirm our previously described hypothesis (presence of ruminant faeces or the in-situ decay of wooden structures or accumulation of forage). The BAs content supports the first hypothesis. The predominance of DCA over LCA and their high ratios (11–68) suggest the attendance of this area by bovines and sheep. Instead, the traces of CDCA, which we found in sample 7 can attribute faecal inputs to another herbivore species, namely goats, whose key skeleton elements are difficult to differentiate from those of sheep and have faeces with a similar morphology^35^, as supported also by the comparison between DCA, CDCA and LCA quantities. In detail, the ratio DCA/CDCA shows a value of 40, not included in the interval 0.8–1.2^35^, the value of the ratio DCA/LCA is not included in the interval 1.0–3.4^35^ and CDCA is not prevalent over DCA. These data confirm the previous study by Prost et al., which rejects the hypothesis of faecal inputs of equine and poultry species and confirm instead goat ejections. Additionally, the circulation of goats, differently from equines and poultry, is supported also by archeozoological studies^42^. However, the great abundance of DCA and the almost absence of CDCA support the prevalence of bovines and the more contained presence of sheep/goats. Finally, the high presence of cholesterol and its metabolites in sample 13 and 14 of the 4-2021 leads to suppose the presence of faecal material derived from omnivore animals, such as dogs and pigs. In samples 10, 11, 12, 13 and 14, the hypothesis of pig ejections is reinforced by the observations of the bile acid UDCA.

### Detection and source identification of faecal matter

As mentioned before, steroid biomarkers have been used in the literature for both a detection of faecal inputs into the environment but also for a source assignment (discussed above).

With the data obtained by our analysis, we were able to apply the ratio from Bull et al.^28^, adopted again by Prost^35^ using the typical human stanols, (coprostanol + epicoprostanol)/(coprostanol + epicoprostanol + 5α-cholestanol). We found a ratio > 0.7 for all the samples, meaning a confirmed faecal imput, except that for sample 8 and 9. These findings, taken together with the detected enhanced contents of bile acids, are clear evidence of faecal input, as bile acids are only produced by vertebrates.

## Conclusions

The study of faecal biomarkers is part of a broader framework of paleoenvironmental investigations; together with archaeozoological and archaeobotanical analyses, it increases our knowledge of how ancient local communities exploited natural resources and may allow us to deduce what their impact on the landscape was. It could furthermore represent a valid tool to better define archaeological deposits related to the presence of domestic animals within the scope of pastoral or agricultural activities. For example, as seems plausible for the Colombare site, it gives the possibility of supposing the presence of areas dedicated to the recovery of animals, with deposits of dung associated to heaps of plant material. But dung and, more generally, droppings have also become a multipurpose reusable resource since prehistoric man began exploiting secondary products of animal origin. Here, we set up and validated the first GC–MS method able to simultaneously identify and quantify (in the range 5–125 pg/mg) BAs and the steroids in soil samples using a fast and simple extraction protocol. Our results are now in agreement with the composition of the faunal population deduced from the remains collected in the 1950s by Zorzi. although the excavation methodology adopted at the time did not allow for an accurate collection of these materials either chronologically or topographically (in fact the archaeological excavation was made by “arbitrary cuts” and a sequence of only three layers was detected and uncovered. These ones, of considerable thickness, contained lithic industry and pottery from different chrono-cultural horizons, so it has been difficult to delineate a punctual relative chronology according to typological criteria. Moreover, no detailed documentation has been preserved that enable archaeologists to understand the close correlation between the finds, including the faunal remains, the squares into which the excavation area was divided, and the structures identified at the time as huts. Riedel, the zooarchaeologist, was forced to study the faunal remains as a single lot in order to give them statistical significance, but he had to forego their chronological and topographical definition). Although the archaeological sediment samples examined are numerically exiguous, the content of faecal biomarkers detected is supported by stratigraphic reliability and radiocarbon dating (some already available, others in progress), so it is and will be possible to outline a concentration trend with stratigraphic detail. The comparative reading with new studies on faunal material collected in the same stratigraphic detail during recent excavation campaigns—including the estimation of sex, age of death and the study of killing curves—will allow to better clarify the economic interest of the animal species reared at the Colombare site (cattle, ovines and pigs) and shed light on the management of livestock farming, also considering the problem of the seasonality. Finally, once a more precise functional characterisation of the areas of the site has been delineated with the continuation of the excavations, they will make it possible to trace the peculiar uses of faecal matter in relation to specific *chaînes opérationnelles*, such as the fertilisation of gardens or the use of dung as building material (dung can also be understood in a broader sense as temper), and to recover much information on the ecological and economic management of a highly potential but complex resource such as excrement by the communities that inhabited the Colombare site.

## Supplementary Information

Supplementary Information.

## Data Availability

All data generated during this study are included in this published article and its supplementary information files.
